# Special Issue on “Advances in Cholesterol and Lipid Metabolism”

**DOI:** 10.3390/metabo12080765

**Published:** 2022-08-19

**Authors:** Allison B. Reiss, Joshua De Leon

**Affiliations:** Department of Medicine, Biomedical Research Institute, NYU Long Island School of Medicine, Mineola, NY 11501, USA

Cholesterol and lipid metabolism is a broad topic that encompasses multiple aspects of cellular function in every organ. Cholesterol and lipids are key constituents of cell membranes that are essential to the brain and, at times, detrimental to the heart. Cholesterol and lipid biosynthesis and transport are tightly regulated and, when dysregulation occurs, the consequences can result in disorders such as obesity, fatty liver, type II diabetes and atherosclerosis [1]. The brain contains approximately 25% of total body cholesterol [2]. Lipid dysregulation can profoundly affect the brain and central nervous system in neurodegenerative disorders such as Alzheimer’s disease and Parkinson’s disease [3]. Abnormal cholesterol and lipid metabolism is a prominent feature of cancer growth and metastasis [4,5]. In addition, chronic kidney disease is associated with premature aging, high cardiovascular risk and cardiometabolic abnormalities [6,7]. Dyslipidemia, abnormal lipoprotein composition, oxidative stress and inflammation may all contribute to the relationship between kidney disease and atherosclerotic cardiovascular disease (ASCVD) [8]. In this Special Issue, we have brought together seminal articles from experts in the field covering novel areas of interest in cholesterol and lipid metabolism and its importance in health and longevity.

Cholesterol is a degradation-resistant hydrophobic molecule that is primarily synthesized in the liver and then secreted into the bloodstream in the form of very-low-density lipoprotein (VLDL), which is subsequently converted to low-density lipoprotein (LDL) [9]. LDL binds to the LDL receptor on the cell surface and is then internalized, entering cells via receptor-mediated endocytosis. LDL receptor gene expression is under negative feedback regulation by intracellular cholesterol levels via sterol regulatory element-binding protein (SREBP) [10]. Atherosclerotic cardiovascular disease (ASCVD) risk is currently determined using the lipid profile with an emphasis on the levels of LDL and high-density lipoprotein (HDL) [11]. Clinical progress in reducing the risk of ASCVD and stroke has occurred as new therapeutic strategies evolve to improve lipid profile, reduce inflammation and, moving forward, enhance the quality of lipoprotein carriers such as HDL. It is increasingly recognized that the concentration of circulating lipoprotein particles alone is not an accurate measure of atherogenic potency; instead, the size, density, number of particles and subclasses are important determinants of risk for plaque formation [12,13]. While current guidelines highlight statins as the mainstay of pharmacological lipid-lowering therapy, newer options and combinations such as fibrates, proprotein convertase subtilisin/kexin type 9 (PCSK9) inhibitors and bempedoic acid are gaining acceptance [14,15].

Our Special Issue encompasses an array of particularly pertinent themes. Gunda et al. [16] combine murine, human and cell culture experiments in a study of cholesterol synthesis in pancreatic adenocarcinoma, one of the most lethal malignancies [17]. 

Turning to the relationship between kidney disease and the high burden of co-morbidities, Vecka and colleagues present a study which compares 26 hemodialysis patients to 26 age- and sex-matched controls and found that the end-stage renal disease patients exhibited higher cholesterol absorption, increased levels of inflammatory markers and an abnormal lipid profile with lower HDL, elevated intermediate density lipoprotein (IDL) and higher numbers of chylomicron remnants [18]. Higher ASCVD incidence may be related to these differences, but further investigation is needed to clarify this relationship. 

Continuing the subject of renal disease and lipid metabolism, Dr. Xiaoyue Pan [19] provides a comprehensive overview of the regulation and function of apolipoproteins in the kidney under normal circumstances and in renal disease states. This review applies a fresh perspective to the individual lipoproteins and how they interact with the kidney. An emphasis is placed on the impact of inflammatory processes that are common in kidney disease and in diabetes where kidney damage and lipid dyshomeostasis co-occur. 

Cui et al. [20] examined the fecal metabolome in healthy older adults using (1H) nuclear magnetic resonance spectroscopy and gas chromatography–mass spectrometry in 83 males and 80 females (age 70 ± 5 years). They found increased concentrations of the amino acids valine, alanine and phenylalanine and in the nucleotide base uracil in those with a higher body mass index. Fecal short-chain fatty acids (SCFAs), commonly produced by the fermentation of indigestible fiber by gut bacteria, were found at concentrations that were lower in those classified as the high-fitness group compared to the low-fitness group [21]. SCFAs are implicated in the regulation of inflammation and risk for ASCVD, making this a new contribution with potential clinical application [22].

It is well-recognized that macrophage reverse cholesterol transport through a pathway involving the activation of ATP-binding cassette transporter (ABC) A1 and ABCG1 prevents lipid-overload and foam cell transformation and is therefore atheroprotective [23]. ABCA1 and ABCG1 are transcriptionally upregulated by the nuclear hormone receptors liver X receptor (LXR) and retinoid X receptor (RXR). This pathway also participates in the control of cholesterol efflux from vascular endothelium where the prevention of cholesterol accumulation is anti-inflammatory and anti-apoptotic [24]. Using cultured immortalized mouse aortic endothelial cells, Huang et al. showed that the upregulation of ABCA1 by specific LXR and RXR agonism not only enhanced ABCA1 expression, but also increased apoAI-mediated cholesterol efflux [25]. They postulate that the delivery of LXR/RXR agonists directly to the endothelium could be anti-atherosclerotic with minimal systemic toxicity.

Our own group has contributed a review of the latest information on apoB and ASCVD [26]. This review highlights that ApoB particles, which represent the sum of atherogenic particles circulating in the plasma, are as important or more important than LDL and non-HDL in determining ASCVD risk [27]. Since each apoB-containing lipoprotein particle contains exactly one molecule of apoB, the number of apoB molecules in circulation reflects the atherogenicity in aggregate and may show superior accuracy, particularly in the setting of type 2 diabetes [28]. 

The consumption of excess sugar is a growing problem that can exacerbate diabetes, contribute to obesity and magnify cardiovascular risk [29,30]. Sugar-sweetened beverages have been linked to dyslipidemia in the Framingham Offspring Study [31]. Busnatu et al. [32] present an interesting perspective on fructose as a risk factor for cardiovascular disease. This paper may spark wide interest as highly processed foods and sweetened beverages containing large amounts of simple sugars are widely consumed and the health consequences of the high-fructose diet are still being explored [33,34,35]. Busnatu et al. address the issue of the COVID-19 pandemic and its adverse effects on activity and diet, making their update particularly timely.

Within this Special Issue of *Metabolites*, it is our aim to present a collection of clinically relevant articles that highlight some of the more recent and less explored avenues of investigation into the cholesterol and lipid metabolism. We spotlight systemic effects of dysregulated lipid metabolism on overall health and on specific organs such as the heart and kidney. As guest editors, we thank all the authors who contributed to this Special Issue, the peer reviewers and the staff of the *Metabolites* Editorial Office for their support and contributions. A very special thank you to Ms. Sherlyn Shui, Section Manager Editor, for her invaluable guidance and advice as we put together this issue. We hope that this Special Issue provides valuable information that can help in medical practice, research efforts and/or everyday life and health.

## References

[1] Luo J., Yang H., Song B.-L. (2020). Mechanisms and regulation of cholesterol homeostasis. Nat. Rev. Mol. Cell Biol..

[2] Björkhem I., Meaney S. (2004). Brain Cholesterol: Long Secret Life Behind a Barrier. Arterioscler. Thromb. Vasc. Biol..

[3] Bian X., Liu R., Meng Y., Xing D., Xu D., Lu Z. (2021). Lipid metabolism and cancer. J. Exp. Med..

[4] Broadfield L.A., Pane A.A., Talebi A., Swinnen J.V., Fendt S.-M. (2021). Lipid metabolism in cancer: New perspectives and emerging mechanisms. Dev. Cell.

[5] Ho W.Y., Hartmann H., Ling S.C. (2022). Central nervous system cholesterol metabolism in health and disease. IUBMB Life.

[6] Jankowski J., Floege J., Fliser D., Böhm M., Marx N. (2021). Cardiovascular Disease in Chronic Kidney Disease: Pathophysiological Insights and Therapeutic Options. Circulation.

[7] Cao H., Meng X. (2022). HDL and Kidney Diseases. Adv. Exp. Med. Biol..

[8] Chen Z., Shrestha R., Yang X., Wu X., Jia J., Chiba H., Hui S.-P. (2022). Oxidative Stress and Lipid Dysregulation in Lipid Droplets: A Connection to Chronic Kidney Disease Revealed in Human Kidney Cells. Antioxidants.

[9] Stellaard F. (2022). From Dietary Cholesterol to Blood Cholesterol, Physiological Lipid Fluxes, and Cholesterol Homeostasis. Nutrients.

[10] Zhou R., Stouffer G.A., Frishman W.H. (2022). Cholesterol Paradigm and Beyond in Atherosclerotic Cardiovascular Disease: Cholesterol, Sterol regulatory element-binding Protein, Inflammation, and Vascular Cell Mobilization in Vasculopathy. Cardiol. Rev..

[11] Nordestgaard B.G., Langlois M.R., Langsted A., Chapman M.J., Aakre K.M., Baum H., Boren J., Bruckert E., Catapano A., Cobbaert C. (2020). Quantifying atherogenic lipoproteins for lipid-lowering strategies: Consensus-based recommendations from EAS and EFLM. Atherosclerosis.

[12] Borén J., Chapman M.J., Krauss R.M., Packard C.J., Bentzon J.F., Binder C.J., Daemen M.J., Demer L.L., Hegele R.A., Nicholls S.J. (2020). Low-density lipoproteins cause atherosclerotic cardiovascular disease: Pathophysiological, genetic, and therapeutic insights: A consensus statement from the European Atherosclerosis Society Consensus Panel. Eur. Heart J..

[13] Suleiman S., Coughlan J.J., Maher V. (2020). Quality over Quantity: A Case Based Review of HDL Function and Dysfunction. Int. J. Clin. Cardiol..

[14] Abdul-Rahman T., Bukhari S.M.A., Herrera E.C., Awuah W.A., Lawrence J., de Andrade H., Patel N., Shah R., Shaikh R., Capriles C.A.A. (2022). Lipid Lowering Therapy: An Era Beyond Statins. Curr. Probl. Cardiol..

[15] Ruscica M., Sirtori C.R., Carugo S., Banach M., Corsini A. (2022). Bempedoic Acid: For Whom and When. Curr. Atheroscler. Rep..

[16] Gunda V., Genaro-Mattos T.C., Kaushal J.B., Chirravuri-Venkata R., Natarajan G., Mallya K., Grandgenett P.M., Mirnics K., Batra S.K., Korade Z. (2022). Ubiquitous Aberration in Cholesterol Metabolism across Pancreatic Ductal Adenocarcinoma. Metabolites.

[17] Andersson R., Haglund C., Seppänen H., Ansari D. (2022). Pancreatic cancer—The past, the present, and the future. Scand. J. Gastroenterol..

[18] Vecka M., Dušejovská M., Staňková B., Rychlík I., Žák A. (2021). A Matched Case-Control Study of Noncholesterol Sterols and Fatty Acids in Chronic Hemodialysis Patients. Metabolites.

[19] Pan X. (2022). The Roles of Fatty Acids and Apolipoproteins in the Kidneys. Metabolites.

[20] Cui M., Trimigno A., Castro-Mejía J.L., Reitelseder S., Bülow J., Bechshøft R.L., Nielsen D.S., Holm L., Engelsen S.B., Khakimov B. (2021). Human Fecal Metabolome Reflects Differences in Body Mass Index, Physical Fitness, and Blood Lipoproteins in Healthy Older Adults. Metabolites.

[21] Deleu S., Machiels K., Raes J., Verbeke K., Vermeire S. (2021). Short Chain Fatty Acids and its Producing Organisms: An Overlooked Therapy for IBD?. eBioMedicine.

[22] Li M., van Esch B., Henricks P., Folkerts G., Garssen J. (2018). The Anti-inflammatory Effects of Short Chain Fatty Acids on Lipopolysaccharide- or Tumor Necrosis Factor α-Stimulated Endothelial Cells via Activation of GPR41/43 and Inhibition of HDACs. Front. Pharmacol..

[23] Voloshyna I., Reiss A.B. (2011). The ABC transporters in lipid flux and atherosclerosis. Prog. Lipid Res..

[24] Xie Q., Peng J., Guo Y., Li F. (2021). MicroRNA-33-5p inhibits cholesterol efflux in vascular endothelial cells by regulating citrate synthase and ATP-binding cassette transporter A1. BMC Cardiovasc. Disord..

[25] Huang K., Jo H., Echesabal-Chen J., Stamatikos A. (2021). Combined LXR and RXR Agonist Therapy Increases ABCA1 Protein Expression and Enhances ApoAI-Mediated Cholesterol Efflux in Cultured Endothelial Cells. Metabolites.

[26] Behbodikhah J., Ahmed S., Elyasi A., Kasselman L.J., De Leon J., Glass A.D., Reiss A.B. (2021). Apolipoprotein B and Cardiovascular Disease: Biomarker and Potential Therapeutic Target. Metabolites.

[27] Zhang C., Ni J., Chen Z. (2022). Apolipoprotein B Displays Superior Predictive Value Than Other Lipids for Long-Term Prognosis in Coronary Atherosclerosis Patients and Particular Subpopulations: A Retrospective Study. Clin. Ther..

[28] Hagström E., Steg P.G., Szarek M., Bhatt D.L., Bittner V.A., Danchin N., Diaz R., Goodman S.G., Harrington R.A., Jukema J.W. (2022). Apolipoprotein B, Residual Cardiovascular Risk After Acute Coronary Syndrome, and Effects of Alirocumab. Circulation.

[29] Yang Q., Zhang Z., Gregg E.W., Flanders W.D., Merritt R., Hu F.B. (2014). Added Sugar Intake and Cardiovascular Diseases Mortality among US Adults. JAMA Intern. Med..

[30] Haslam D.E., Chasman D.I., Peloso G.M., Herman M.A., Dupuis J., Lichtenstein A.H., Smith C.E., Ridker P.M., Jacques P.F., Mora S. (2022). Sugar-sweetened Beverage Consumption and Plasma Lipoprotein Cholesterol, Apolipoprotein, and Lipoprotein Particle Size Concentrations in U.S. Adults. J. Nutr..

[31] Lee Y.Q., Whitton C., Neelakantan N., van Dam R.M., Chong M.F.-F. (2022). Dietary patterns and predicted 10-year cardiovascular disease risk in a multiethnic Asian population. Nutr. Metab. Cardiovasc. Dis..

[32] Busnatu S.-S., Salmen T., Pana M.-A., Rizzo M., Stallone T., Papanas N., Popovic D., Tanasescu D., Serban D., Stoian A.P. (2022). The Role of Fructose as a Cardiovascular Risk Factor: An Update. Metabolites.

[33] Chan L.Y., Coyle D.H., Wu J.H.Y., Louie J.C.Y. (2021). Total and Free Sugar Levels and Main Types of Sugars Used in 18,784 Local and Imported Pre-Packaged Foods and Beverages Sold in Hong Kong. Nutrients.

[34] Tappy L. (2018). Fructose-containing caloric sweeteners as a cause of obesity and metabolic disorders. J. Exp. Biol..

[35] Hieronimus B., Medici V., Bremer A.A., Lee V., Nunez M.V., Sigala D.M., Keim N.L., Havel P.J., Stanhope K.L. (2020). Synergistic effects of fructose and glucose on lipoprotein risk factors for cardiovascular disease in young adults. Metabolism.

